# Chemical Signals and Mechanosensing in Bacterial Responses to Their Environment

**DOI:** 10.1371/journal.ppat.1005057

**Published:** 2015-08-27

**Authors:** Akshay K. Harapanahalli, Jessica A. Younes, Elaine Allan, Henny C. van der Mei, Henk J. Busscher

**Affiliations:** 1 University of Groningen and University Medical Center Groningen, Department of Biomedical Engineering, Groningen, The Netherlands; 2 Division of Microbial Diseases, UCL Eastman Dental Institute, University College London, London, United Kingdom; University of North Carolina at Chapel Hill School of Medicine, UNITED STATES

Bacteria encounter different environmental conditions during the course of their growth and have developed various mechanisms to sense their environment and facilitate survival. Bacteria are known to communicate with their environment through sensing of chemical signals such as pH, ionic strength, or sensing of biological molecules, as utilized in quorum sensing [1]. However, bacteria do not solely respond to their environment by means of chemical sensing, but also respond through physical-sensing mechanisms. For instance, upon adhesion to a surface, bacteria may respond by excretion of extracellular-polymeric-substances (EPS) through a mechanism called mechanosensing, allowing them to grow in their preferred, matrix-protected biofilm mode of growth [2]. Chemical sensing of antimicrobials may further enhance EPS excretion [3]. We will now first discuss the distinction between chemical- and mechanosensing mechanisms and subsequently elaborate further on mechanosensing.

## What Distinguishes Chemical Sensing from Mechanosensing?

Chemical sensing relies on the presence of specific molecules such as H^+^ ions, antimicrobials, or on the presence of excreted biological signaling molecules that need to diffuse toward neighbouring organisms to enable communication and response. In general, gram-negative bacteria use homoserine lactones as signaling molecules [4], while peptides are predominantly used by gram-positive bacteria [5]. When signaling molecules have reached a threshold concentration, they activate a receptor that induces expression of target genes to control the response.

In mechanosensing, bacteria are required to come into physical contact with their environment, for instance by adhering to a substratum surface or the surfaces of neighbouring bacteria. This can either be through nonspecific or highly specific ligand–receptor interactions (see also below). Some bacterial cells have special surface appendages, like flagella or pili that can come in direct, physical contact with another surface. In *Vibrio parahaemolyticus*, for instance, physical contact can act as a signal, to switch the population from a planktonic to a sessile, surface-adhering phenotype [6]. *Vibrio cholerae* can use its flagellum as a mechanosensor, and upon contact with a hard surface, the flagellar motor stops and ion flow through the motor ceases, which increases the membrane potential and initiates biofilm formation [7].

Not all bacterial strains possess surface appendages to probe a surface, yet upon adhesion to a surface they respond by producing EPS and adapting a biofilm mode of growth. Another form of mechanosensing of a surface is based on adhesion force–induced deformation of the bacterial cell wall. In *Staphylococcus aureus*, adhesion forces to substratum surfaces have been found to modulate *icaA* expression and associated EPS production [8]. Moreover, adhesion force–modulated *icaA* expression was disturbed in mutants lacking a rigid, cross-linked peptidoglycan layer, suggesting that this form of mechanosensing depends on an intricate balance between rigidity of the bacterial cell wall and prevailing adhesion forces. The lipid membrane subsequently follows the deformation of the more rigid peptidoglycan layer in the cell wall.

## How Does Cell Wall Deformation Yield Surface Sensing?

**Fig 1:**
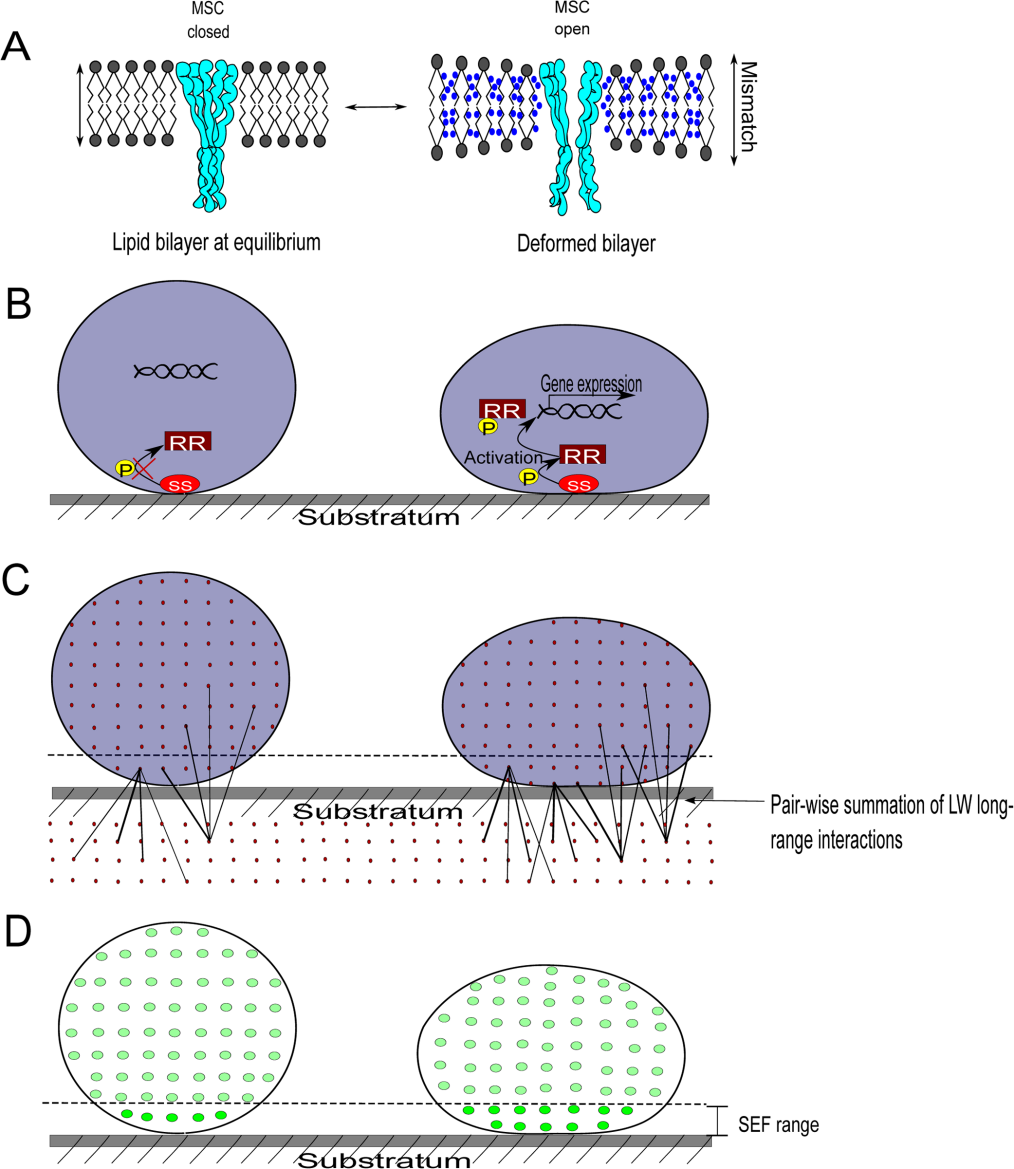
Bacterial cell wall deformation, mechanosensing, and the measurement of cell wall deformation using surface enhanced fluorescence. **A) Left:** Intact lipid membrane at equilibrium of an undeformed bacterium, with a closed mechanosensitive channel (MSC). **Right:** Bacterium adhering to a substratum surface, deformed under the influence of adhesion forces arising from the substratum, yielding hydrophobic mismatch over the thickness of the membrane (water molecules adjacent to hydrophobic lipid tails), and altered lipid bilayer tension in the lipid membrane. Hydrophobic mismatch and pressure profile changes lead to the opening of MSCs. **B) Left:** A nonactivated stress-sensitive (SS) protein on the bacterial cell surface of an undeformed bacterium and a response regulator protein (RR) suspended freely in the cytoplasm. **Right:** A SS protein senses cell wall deformation due to adhesion, changes its conformation, and phosphorylates a RR protein which regulates the expression of SS-regulated genes. **C) Left:** Lifshitz-Van der Waals forces operate between all molecular pairs in a bacterium and a substratum, decreasing with distance between the molecules. **Right:** Adhering bacterium, deformed due to attractive Lifshitz-Van der Waals forces, with more molecules in the bacterium closer to the substratum, yielding stronger adhesion and more deformation. Deformation stops once the counterforces arising from the deformation of the rigid peptidoglycan layer match those of the adhesion forces. **D) Left:** Only a small number of fluorophores inside an undeformed bacterium are sufficiently close to a metal substratum surface to experience surface-enhanced fluorescence (brighter dots). **Right:** In a deformed, adhering bacterium, the volume of the bacterium close to the surface increases and the number of fluorophores subject to surface-enhanced fluorescence becomes higher. Thus, quantitative analysis of fluorescence arising from fluorescent bacteria adhering to a metal surface provides a way to determine cell wall deformation.

When a bacterial cell wall deforms either under the influence of adhesion forces arising from a substratum surface or due to other external forces, the intrabilayer pressure profile across the lipid membrane changes as a result of bilayer deformation [9]. Pressure profile changes can be sensed by bacteria in two different ways: one is through a physical approach (gating of the mechanosensitive channel, see Fig 1A) and the other through responses generated by stress-sensitive (SS) proteins on the cell surface (Fig 1B).

Cell wall deformation occurs at the expense of energy, provided by the adhesion forces arising from the substratum surface to which bacteria adhere. This energy is required to compensate for the energetically unfavorable contact between hydrophobic membrane lipids and water (“hydrophobic mismatch”) and the geometric consequences (thinning of the lipid membrane and wider spacing between lipid molecules) of the lipid bilayer intrinsic curvature (Fig 1A) [9]. Membrane-intrinsic curvature changes in *Escherichia coli* were found to trap membrane channels in a fully open state, while hydrophobic mismatch alone was unable to open channels. Accordingly, mechanosensitive channels must be considered as interpreters of membrane tension [10] through which mechanical stimuli can be translated into a biological response. Similarly, SS proteins present on the cell surface can become activated upon cell wall deformation. In the Cpx two-component system in *E*. *coli*, for example [11], the SS protein CpxA can autophosphorylate and transfer phosphate groups to the response regulator protein CpxR in the cytoplasm. Subsequently, the phosphorylated CpxR binds to multiple regulatory sites of the DNA to increase transcription of target genes.

## How Can We Experimentally Demonstrate and Quantify Bacterial Cell Wall Deformation upon Adhesion to Surfaces?

Bacterial adhesion to surfaces is mediated by adhesion forces arising from the substratum surface to which they adhere. From a physicochemical perspective, there are only a limited number of different adhesion forces:

- Lifshitz-Van der Waals forces, generally attractive and operative over a relatively long distance range;
- electrostatic forces that can either be attractive or repulsive depending on their magnitude and distance range, as determined by ionic strength and pH;
- acid–base interactions between hydrogen-donating and hydrogen-accepting groups that can also be attractive or repulsive.

When these adhesion forces arise from spatially localized and stereochemical groups, they are sometimes called “specific,” or ligand–receptor interactions [12].

Due to the long-range nature of Lifshitz-Van der Waals forces, contributions to the total Lifshitz-Van der Waals force arise from all molecular pairs in a bacterium and a substratum, which of course decrease in magnitude with increasing distance (Fig 1C) [13]. It has been argued that, since the overall molecular composition of different bacterial strains is highly similar, differences in Lifshitz-Van der Waals forces between adhering bacteria on different substratum surfaces reflect varying degrees of cell wall deformation. The rationale for this is simple: deformation brings more molecules in the close vicinity of a substratum, average distance will decrease and adhesion forces increase, yielding more extensive deformation until impeded by counterforces arising from the rigidity of the peptidoglycan layer. It is uncertain whether ligand–receptor interactions can also mediate cell wall deformation to the extent as nonspecific Lifshitz-Van der Waals forces have been demonstrated to do [14]. Since ligand–receptor interactions only arise from molecules present at the surface, their number is small as compared to the number of molecules participating in Lifshitz-Van der Waals forces (see Fig 1C). However, their strength of interaction may be quite strong.

Adhesion-induced cell wall deformation has been directly demonstrated through atomic force microscopy measurements of the height and base width of bacteria adhering to substratum surfaces, but atomic force microscopy data have to be obtained for individual bacteria, which is a tedious procedure with high variability [14]. As an alternative method to quantify bacterial cell wall deformation, surface-enhanced fluorescence has been proposed. Surface-enhanced fluorescence is based on recent observations that fluorescence is enhanced on reflecting surfaces once the fluorophores are within the range of 20–30 nm from the surface [15]. Similarly, upon adhesion of fluorescent bacteria to a reflecting surface, cell wall deformation will occur that brings a larger volume of the bacterium and therewith more fluorophores closer to a surface, yielding stronger surface-enhanced fluorescence (Fig 1D). Surface-enhanced fluorescence of adhering bacteria can be measured using macroscopic bio-optical imaging that allows observation over substratum areas of several tens of cm^2^, therewith encompassing numbers of adhering bacteria that approximate a bacterial monolayer (around 10^8^ bacteria/cm^2^). Accordingly, surface-enhanced fluorescence has been proposed as an ideal method to study adhesion-induced cell wall deformation in a rapid and statistically reliable manner under naturally occurring adhesion forces, the only drawback being the need to use a reflecting surface and fluorescent bacterial strains.

## Does Physical Contact between Bacteria Modulate Quorum Sensing?

Physical contact is not only established between bacteria adhering to substratum surfaces but also between individual bacteria in a biofilm, which raises a number of interesting questions. First of all, biofilms produce different amounts of EPS depending on the nature of the substratum [3], but only the initially adhering bacteria have contact with the substratum surface itself [16]. Clearly, the effective range of all attractive or repulsive forces arising from a substratum surface is limited to tens of nanometres, making it impossible for bacterial cells other than the initial colonisers to directly sense a surface. Moreover, they will experience adhesion forces from neighboring organisms with whom they coadhere. This implies that there must be a communication means available within a biofilm through which substratum information is passed to bacteria that are not in direct contact with the substratum enabling them to indirectly sense the surface. Quorum sensing likely is the prevailing mechanism for the indirect passing of this information to later colonizers in a biofilm, although physical contact between coadhering bacteria may play a role here too. For instance, *Myxococcus xanthus*, *E*. *coli*, *Bacillus subtilis*, and lactobacilli use contact-dependent signaling for communication [17] in addition to quorum sensing, suggesting that physical contact not only provides a direct way of communication between bacteria within their environment; moreover it may also constitute a mechanism by which bacteria can optimise the use of quorum-sensing molecules. For example, lactobacilli adhere more strongly to staphylococci than staphylococci to each other, giving lactobacilli the opportunity to penetrate and colonise regions of vaginal biofilms where staphylococci predominate, resulting in the quorum-sensing–mediated quenching of staphylococcal toxic shock syndrome toxin secretion [18,19]. This form of quorum quenching only occurs however, when there is a sufficiently high concentration of quorum-quenching dipeptides in the close neighborhood of toxic shock syndrome toxin-secreting staphylococci, which occurs more readily when staphylococci and lactobacilli are in direct contact with each other [18]. Thus physical contact, as established through adhesion forces between bacteria and biochemical signaling, may be considered as intrinsically linked mechanisms in a biofilm.

## Perspective: Cell Wall Deformation and Adhesion-Induced Antibiotic Resistance of Biofilms

Eighty percent of all human infections are caused by biofilms adhering to soft tissue surfaces in the human body, the surfaces of biomaterial implants or coadhering to other bacteria. The antibiotic resistance of biofilms exceeds that of planktonic bacteria [20] due to phenotypic changes induced by adhesion of the bacteria involved and their production of an EPS matrix, which hampers antimicrobial penetration [21]. Cell wall deformation induced by adhesion forces plays a pivotal role in this transition from antibiotic-susceptible planktonic growth to a more antibiotic-resistant biofilm mode of growth, and production of a protective EPS matrix has been found absent for bacteria adhering to surfaces exerting weak adhesion forces [22]. However, this implies only indirect evidence for the involvement of mechanosensitive channels or SS proteins in bacterial biofilm formation. Therefore, control of the forces experienced by bacteria in a biofilm may provide a relatively unexplored pathway to control resistance associated with implant-associated infections and perhaps the pathogenicity of biofilms.
